# Effect of Acetaldehyde Intoxication and Withdrawal on NPY Expression: Focus on Endocannabinoidergic System Involvement

**DOI:** 10.3389/fpsyt.2014.00138

**Published:** 2014-10-01

**Authors:** Fulvio Plescia, Anna Brancato, Rosa Anna Maria Marino, Carlotta Vita, Michele Navarra, Carla Cannizzaro

**Affiliations:** ^1^Department of Sciences for Health Promotion and Mother and Child Care “Giuseppe D’Alessandro”, University of Palermo, Palermo, Italy; ^2^Department of Drug Sciences and Products for Health, University of Messina, Messina, Italy

**Keywords:** acetaldehyde withdrawal, neuropeptide Y expression, endocannabinoidergic system, hippocampus, nucleus accumbens

## Abstract

Acetaldehyde (ACD), the first alcohol metabolite, plays a pivotal role in the rewarding, motivational, and addictive properties of the parental compound. Many studies have investigated the role of ACD in mediating neurochemical and behavioral effects induced by alcohol administration, but very little is known about the modulation of neuropeptide systems following ACD intoxication and withdrawal. Indeed, the neuropeptide Y (NPY) system is altered during alcohol withdrawal in key regions for cerebrocortical excitability and neuroplasticity. The primary goal of this research was to investigate the effects of ACD intoxication and withdrawal by recording rat behavior and by measuring NPY immunoreactivity in hippocampus and NAcc, two brain regions mainly involved in processes which encompass neuroplasticity in alcohol dependence. Furthermore, on the basis of the involvement of endocannabinoidergic system in alcohol and ACD reinforcing effects, the role of the selective CB1 receptor antagonist AM281 in modulating NPY expression during withdrawal was assessed. Our results indicate that (i) ACD intoxication induced a reduction in NPY expression in hippocampus and NAcc; (ii) symptoms of physical dependence, similar to alcohol’s, were scored at 12 h from the last administration of ACD; and (iii) NPY levels increased in early and prolonged acute withdrawal in both brain regions examined. The administration of AM281 was able to blunt signs of ACD-induced physical dependence, to modulate NPY levels, and to further increase NPY expression during ACD withdrawal both in hippocampus and NAcc. In conclusion, the present study shows that complex plastic changes take place in NPY system during ACD intoxication and subsequent withdrawal in rat hippocampal formation and NAcc. The pharmacological inhibition of CB1 signaling could counteract the neurochemical imbalance associated with ACD, and alcohol withdrawal, likely boosting the setting up of homeostatic functional recovery.

## Introduction

Acetaldehyde (ACD), the first oxidation product of alcohol, is one of the mediators of the peripheral and central effects of alcohol (1–5), in particular playing a main role in the rewarding, motivational, and addictive properties of the parental compound (6–8). Although not fully investigated, ACD reinforcing properties are likely due to its capability to affect the dopaminergic and endocannabinoidergic systems. As alcohol, ACD is able to induce and maintain an operant drinking behavior and relapse following repeated forced abstinence (6) and to increase the firing rate, spikes/burst, and burst firing of VTA neurons (9–11); furthermore, the pharmacological manipulation of dopaminergic D2 and endocannabinoidergic CB1 receptors decreases its motivational and incentive value (8, 12). As largely described, after abrupt suspension of long-term repetitive consumption, alcohol withdrawal syndrome reflects severe neuro-adaptation of membrane and intracellular molecular targets that results in disruption and perturbation in neurotransmitter and neuropeptide systems (13–15). Among them, CRH and neuropeptide Y (NPY) have been mainly evoked as responsible for the affective and somatic components of alcohol withdrawal (16, 17). In particular, NPY, a 36-amino acid peptide neuromodulator largely distributed in the central nervous system, is implicated in a wide range of functions including feeding, anxiety, seizures, circadian rhythms, memory, and cardiovascular regulation (18–21), besides its involvement in the neuronal mechanisms of alcohol consumption (22, 23). Indeed, lower levels of NPY-IR in hippocampus, amygdala, and frontal cortex have been reported in selectively bred alcohol-preferring rats compared to non-preferring rats (24), as well as lower expression in NPY protein in NAcc has been measured in C57BL/6J mice, that innately consume larger amount of alcohol (25). On the other hand, intracerebroventricular infusion of NPY produces electrophysiological effects similar to those of alcohol in rats (26); consistently, NPY-deficient mice drink more alcohol compared with wild-type mice, whereas mice over-expressing NPY display a lower preference for alcohol (27, 28). Additionally, NPY plays a central role in the modulation of neuronal excitability mainly in cortex and hippocampus (29–32), where NPY is mostly co-localized with γ-aminobutyric acid within interneurons (33–35). Cerebrocortical excitability is altered during the development of alcohol tolerance and dependence, and greatly enhanced during alcohol withdrawal (36, 37). Notably, intracerebroventricular administration of NPY attenuates symptoms of alcohol withdrawal in rats, probably due to presynaptical inhibition of glutamate release (29, 38, 39). Recent data have identified NPY as a promoter of hippocampal neurogenesis since it is able to enhance cell proliferation and promote neuronal differentiation in adult mice (40–42). Adult neurogenesis occurs constitutively in the subgranular zone of the hippocampal dentate gyrus and in the subventricular zone of the walls of the lateral ventricles, adjacent to the ventral striatum (43–45). The modulation of NPYergic system seems to be involved in the regulation of alcohol-induced reactive neurogenesis (46, 47). Thus, the assessment of ACD activity on NPY expression in brain areas closely linked to the neurogenic niches may be helpful to clarify its role as a mediator of alcohol effects on brain neuroplasticity. In this study, ACD was administered according to a binge model previously characterized to induce tolerance and physical dependence to alcohol (48–51). The effects of ACD intoxication and withdrawal were investigated by recording rat behavior, and by measuring NPY expression in the hippocampus and ventral striatum, two of the brain regions mainly involved in processes which encompass neuroplasticity in alcohol dependence (46). Moreover, since the endocannabinoidergic system plays a relevant role in the reinforcing effects of alcohol (52–55), and also in the development of alcohol tolerance and withdrawal (56), the present research aimed at the evaluation of the effect of a selective CB1 receptor antagonist AM281 on NPY expression during withdrawal. Indeed, an interplay between endocannabinoidergic system and NPY expression and release has been demonstrated in the hypothalamus (57, 58), but so far, no data exist on a functional correlation between ACD, NPY, and endocannabinoids in the brain.

## Materials and Methods

### Animals

Adult male Wistar rats, weighing 250–300 g, were used in this study. Animals were housed two per cage and maintained on a 12 h light/dark cycle, on temperature (22 ± 2°C) and humidity (55 ± 5%) controlled conditions, with *ad libitum* access to food and water. All efforts were made to minimize suffering and number of animals used. Experimental procedures were in strict accordance with Italian legislation dealing with research on experimental animals (D.L. 116/92) and European Council Directive (2010/63/EU) on animals used for scientific purposes.

### Drugs and pharmacological treatment

Acetaldehyde 99.98% (Sigma-Aldrich, Milan, Italy) was daily diluted with tap water to a final concentration of 8% v/v; each intragastric ACD administration provided 450 mg/kg. The selective CB1 receptor antagonist AM281 (Sigma-Aldrich, Milan, Italy) was suspended in saline solution containing 3% Tween 80 and administered i.p. at 2.5 mg/kg, in ACD and CTR animals at day 5, 3 and 12 h after the last intragastric administration [modified from Ref. (59)]. The dose of AM281 used in this study was chosen to avoid any aspecific effect (60, 61).

### Binge ACD treatment

Rats received intragastric infusions of ACD (450 mg/kg) by gavage, five times daily (7 a.m., 11 a.m., 3 p.m., 7 p.m., and 11 p.m.) for 4 days, in order to induce intoxication and withdrawal syndrome (49). The control group received intragastric infusions of water, according to the time schedule of the protocol. Behavioral signs of ACD intoxication were observed according to the severity scale of Majchrowicz (49). During the intoxication paradigm, ACD treatment was individually adjusted to reach an intoxication score between 3 and 5 (Ataxia 2–LRR) on each entire day (51) to avoid lethal toxicity (seven animals died during the protocol, before the assessments.).

Acetaldehyde-binge treated animals and controls were allocated in the following experimental groups according to the assigned procedure: ACD/T1 (*n* = 6) and CTR/T1 (*n* = 6) were decapitated 1 h after the last intragastric administration; ACD/T16 (*n* = 6), CTR/T16 (*n* = 6), ACD + AM281/T16 (*n* = 6), and CTR + AM281/T16 (*n* = 6) were evaluated for the behavioral signs of withdrawal at T12, and were decapitated at 16 h after the last intragastric infusion; ACD/T72 (*n* = 6), CTR/T72 (*n* = 6), ACD + AM281/T72 (*n* = 6), and CTR + AM281/T72 (*n* = 6) were decapitated 72 h after the last intragastric administration.

### Behavioral observations of withdrawal

At 12 h after the last infusion of ACD (T12), an observer blinded to the treatment assessed the severity of physical dependence considering the following signs: general hyperactivity, irritability, tail tremors, tail stiffness, general tremor, spasticity, wet (dog) shakes, and spontaneous convulsive seizures (62). Each sign was assigned a score of 0–3 (0 = not present, 1 = slight, 2 = moderate, and 3 = severe). The sum of these scores (0–24) was used as a quantitative measurement of the severity of the withdrawal reaction, the “total withdrawal score.”

### Immunohistochemical detection of NPY

After decapitation, brains were rapidly removed, frozen on dry ice, and stored at −80°C. Coronal serial sections (20 μm) from frozen rat brains were cut on a cryomicrotome from plate 29 to 36, corresponding to dorsal hippocampus, and from plate 10 to 14, equivalent to nucleus accumbens, according to the atlas of Paxinos and Watson (63). Sections were thaw-mounted onto Superfrost glass slides, dried on a hotplate, and processed for NPY immunohistochemical analysis using a commercially available NPY immunohistochemistry staining kit (D.B.A., Italy). The total number of NPY-positive neurons in each target brain region was achieved by counting the number of positive cells of labeled cell bodies determined with cresyl violet staining. Coronal brain sections were further divided into different quadrants: hippocampus in CA1, CA2, CA3, and DG, nucleus accumbens in shell and core. The counterstained sections were placed under the microscope, and the number of positive cells was counted manually in all quadrants. Each labeled cell was viewed under bright-field illumination using a 100× objective (Meiji Techno, Japan). Real-time microscopic images were captured by a video camera, digitized, and displayed on a monitor. Two repeated measurements by two different experimenters were performed bilaterally in three adjacent sections per animal in the brain regions of interest (63).

### Statistical analysis

The differences in total withdrawal score between the groups were assessed by the Kruskal–Wallis analysis of variance (ANOVA) followed by the Dunn *post hoc* test. A two-way ANOVA was conducted on the number of NPY-positive neurons as dependent variables, with treatment (control, ACD, AM281) as the between-subjects factor and “time” or “brain Area” as within-subjects factor. When necessary, simple main effects and *post hoc* comparison were calculated with Bonferroni post-test (α = 5). Values were considered statistically significant when *p* < 0.05. All data are presented as mean (S.D.). Statistical analysis was conducted by using a GraphPad Prism software 6.1 (GraphPad Software, San Diego, CA, USA) on data from all experimental animals used.

## Results

### Behavioral observations of withdrawal

**Figure 1:**
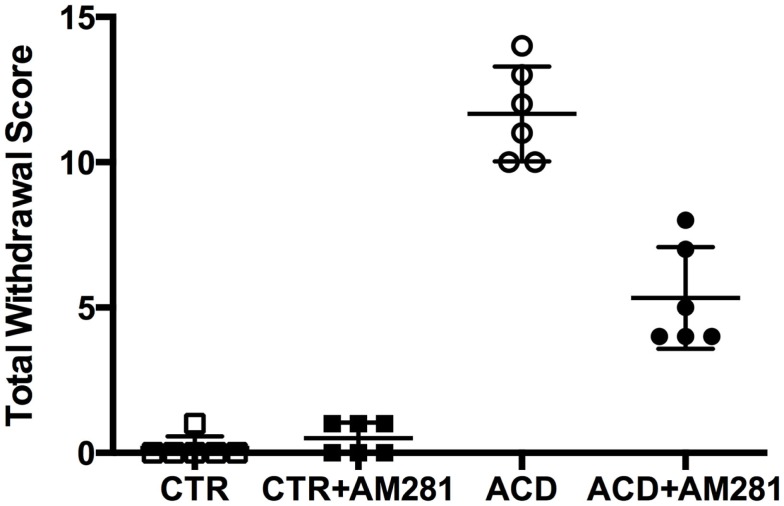
**Total withdrawal score**. (□) CTR, (■) CTR + AM281, (○) ACD, and (●) ACD + AM281.

In order to investigate spontaneous withdrawal behavior, rats were observed at 12 h from the last ACD intragastric administration and scored for general hyperactivity, irritability, tail tremors, tail stiffness, general tremor, spasticity, wet (dog) shakes, and spontaneous convulsive seizures. ACD-treated animals showed discrete behavioral signs of withdrawal, and among them, general hyperactivity, irritability, and spasticity were recorded more frequently. Somatic dependence symptoms persisted until 16 h and were absent when the animals were observed again at 36 h. According to the score assigned to each behavioral sign, the mean total withdrawal score in ACD rats was of 11.67 ± 1.63 (Figure 1). Results of a Kruskal–Wallis test, performed on each behavioral score and on total withdrawal score, including “treatment” as the between-subjects factor, showed significant differences among the experimental groups (general hyperactivity: *p* < 0.00; irritability: *p* < 0.001; tail tremors: *p* < 0.01; tail stiffness: *p* < 0.001; general tremor: *p* < 0.01; spasticity: *p* < 0.001). Dunn’s *post hoc* analysis, highlighted a significantly higher presence of individual and total withdrawal symptoms in ACD group, with respect to CTR, while individual and total withdrawal score of ACD-AM281 rats was non-statistically different than controls’. Results of Dunn’s *post hoc* analysis are showed in Table 1.

**Table 1:** **Behavioral observations of withdrawal**.

Withdrawal behaviors	CTR	CTR + AM281	ACD	ACD + AM281
General hyperactivity	0.17 ± 0.41	0.33 ± 0.52	2.55 ± 0.55**	1.67 ± 0.52
Irritability	0	0.17 ± 0.41	2.83 ± 0.41**	1.17 ± 1.17
Tail tremors	0	0	1.00 ± 0.63*	0.50 ± 0.84
Tail stiffness	0	0	1.67 ± 0.51**	1.00 ± 0.89
General tremor	0	0	1.50 ± 1.05**	0.33 ± 0.52
Spasticity	0	0	2.17 ± 0.75**	0.67 ± 0.82
Wet (dog) shakes	0	0	0	0
Spontaneous convulsive seizures	0	0	0	0
Total withdrawal score	0.17 ± 0.41	0.5 ± 0.55	11.67 ± 1.63***	5.33 ± 1.75

Each sign was assigned a score of 0–3 (0 = not present, 1 = slight, 2 = moderate, 3 = severe). The sum of these scores was used as a quantitative measurement of the severity of the withdrawal reaction, the “total withdrawal score.” *p < 0.05; **p < 0.01; ***p < 0.001 vs CTR.

### Quantification of NPY-positive neurons

#### Hippocampus

**Figure 2:**
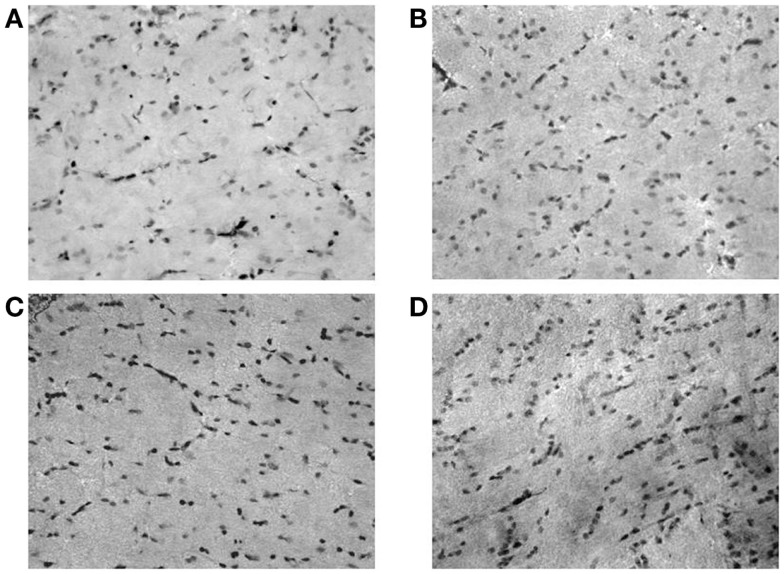
**Microphotographs of neuropeptide Y (NPY)-positive neurons in rat hippocampus (dentate gyrus); (A) controls; (B) ACD rats at T1; (C) ACD rats at T16; and (D) ACD rats at T72**. The specific labeling is observed under bright-field illumination using a 100× objective.

**Figure 3:**
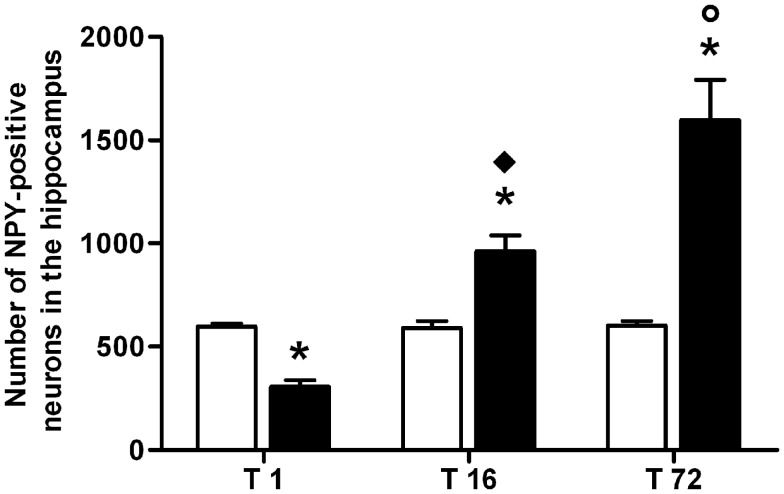
**Average number of neuropeptide Y (NPY)-positive neurons in the hippocampus of ACD and control rats at different times following the last intragastric infusion**. Each value represents the mean ± S.D. of 12 sections for each experimental condition. (□) CTR, (■) ACD. *p < 0.001 vs CTR, ^♦^*p* < 0.001 vs T1, ^○^*p* < 0.001 vs T16.

The number of NPY-positive neurons was evaluated in the hippocampus as a whole. The results of a two-way ANOVA including “ACD treatment” as the between-subjects factor and “time” as within-subjects factor showed a significant effect of time, treatment, and their interaction [*F*_(2, 66)_ = 223.65, *p* < 0.0001; *F*_(1, 66)_ = 204.16, *p* < 0.0001; *F*_(2, 66)_ = 220.28, *p* < 0.0001]. Bonferroni *post hoc* analysis showed a significant reduction in NPY-positive neurons in ACD group at T1 (*t* = 6.729, *p* < 0.001) and an increase in NPY-positive neurons at T16 (*t* = 8.526, *p* < 0.001) and T72 (*t* = 22.95, *p* < 0.001) when compared to CTR group. NPY expression was also analyzed within ACD group in order to reveal time-related differences in relative NPY-positive neurons expression during withdrawal (Figure 2). The results of a two-way ANOVA showed a significant effect of time, treatment, and their interaction [*F*_(2, 66)_ = 233.09, *p* < 0.0001; *F*_(1,66)_ = 212.78, *p* < 0.0001; *F*_(2, 66)_ = 229.57, *p* < 0.0001]. Bonferroni *post hoc* analysis showed that NPY expression increased at T16 (*t* = 15.41, *p* < 0.001), with respect toT1, and at T72 (*t* = 15.01, *p* < 0.001) compared to T16 (Figure 3). A detailed analysis of NPY expression in the hippocampal sub-regions was also carried out. The results of a two-way ANOVA performed, respectively, at T1, at T16, and at T72, including “ACD treatment” as the between-subjects factor and “sub-regional NPY expression” as within-subjects factor showed a significant effect of sub-regional NPY expression [*F*_(3, 88)_ = 1768.82, *p* < 0.0001; *F*_(3,88)_ = 233.30, *p* < 0.0001; *F*_(3, 88)_ = 24.30, *p* < 0.0001], treatment [*F*_(1, 88)_ = 1284.10, *p* < 0.0001; *F*_(1,88)_ = 160.9, *p* < 0.0001; *F*_(1,88)_ = 636.3, *p* < 0.0001] and their interaction [*F*_(3,88)_ = 298.08, *p* < 0.0001; *F*_(3,88)_ = 23.75, *p* < 0.0001; *F*_(2,88)_ = 54.12, *p* < 0.0001). Bonferroni *post hoc* analysis showed a decrease in the number of NPY-positive cells at T1 in ACD group in all the hippocampal sub-regions, while an increase in NPY expression was observed at T16 and T72, compared to controls (Table 2).

**Table 2:** **Number of NPY-positive neurons in different sub-regions of the hippocampus, in ACD and in control rats, at different times following the last intragastric infusion (T1, T16, T72)**.

Time	Hippocampus sub-regions	CTR	ACD	Statistic	
				*t*	*p*
T1	CA1	184 ± 4.3	109 ± 11.0	23.05	<0.001
	CA2	236 ± 6.2	106 ± 9.3	39.82	<0.001
	CA3	53 ± 3.4	48 ± 4.8	1.56	<0.05
	DG	45 ± 13.0	21 ± 6.2	7.35	<0.001
T16	CA1	182 ± 36.0	406 ± 80.0	12.14	<0.001
	CA2	233 ± 46.0	381 ± 76.0	8.023	<0.001
	CA3	52 ± 10.3	66 ± 12.3	1.02	>0.05
	DG	24 ± 4.2	103 ± 22.0	4.445	<0.001
T72	CA1	185 ± 36.0	460 ± 72.0	12.07	<0.001
	CA2	237 ± 49.3	313 ± 53.0	3.511	<0.01
	CA3	56 ± 10.2	331 ± 69.3	12.07	<0.001
	DG	25 ± 2.9	491 ± 77.1	21.53	<0.001

*Values are mean ± S.D. of 12 sections for each experimental condition. CTR, controls; ACD, acetaldehyde; DG, dentate gyrus*.

#### Effects of AM281 on hippocampal NPY expression

**Figure 4:**
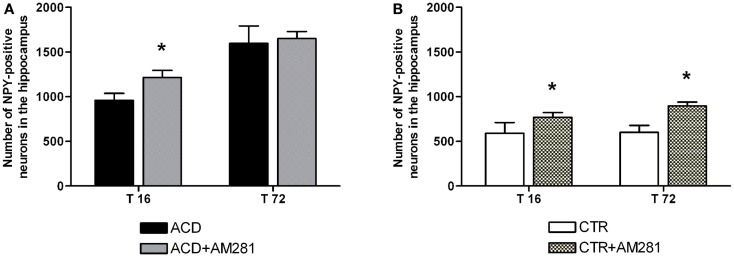
**Effect of AM281 treatment on the number of hippocampus neuropeptide Y (NPY)-positive neurons in ACD rats (A) and in controls (B)**. Each value represents the mean ± S.D. of 12 sections for each experimental condition. *p < 0.001 vs respective controls.

In order to assess the involvement of CB1 signaling on the modulation of NPY-positive neurons expression in hippocampus, statistical analysis by a two-way ANOVA was performed on the effect of the CB1 antagonist AM281 both in ACD group and in controls. Our results showed significant effects of time, treatment, and their interaction in ACD rats [*F*_(1, 44)_ = 422.31, *p* < 0.0001; *F*_(1,44)_ = 34.86, *p* < 0.0001; *F*_(1, 44)_ = 15.29, *p* < 0.0001]. Bonferroni *post hoc* analysis showed that AM281 was able to induce an increase in the number of NPY-positive neurons at 16 h (t = 6.940, *p* < 0.001) in ACD group compared to respective controls (Figure 4A). In control animals, a two-way ANOVA including “AM281 treatment” as the between-subjects factor and “time” as within-subjects factor revealed a significant effect of time, treatment, and their interaction [*F*_(1, 44)_ = 9.24, *p* < 0.0040; *F*_(1,44)_ = 106.99, *p* < 0.0001; *F*_(1, 44)_ = 6.32, *p* < 0.0156]. Bonferroni *post hoc* analysis showed that AM281 was able to induce an increase in the number of NPY-positive neurons at T16 (*t* = 5.536, *p* < 0.001) and T72 (*t* = 9.092, *p* < 0.001) in CTR group (Figure 4B).

#### Nucleus accumbens

**Figure 5:**
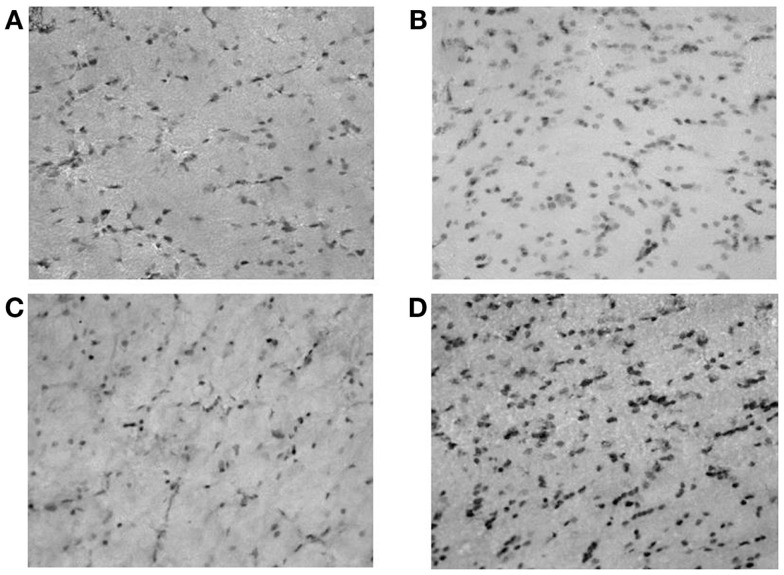
**Microphotographs of neuropeptide Y (NPY)-positive neurons in rat nucleus accumbens (shell); (A) controls; (B) ACD rats at T1; (C) ACD rats at T16; and (D) ACD rats at T72**. The specific labeling is observed under bright-field illumination using a 100× objective.

**Figure 6:**
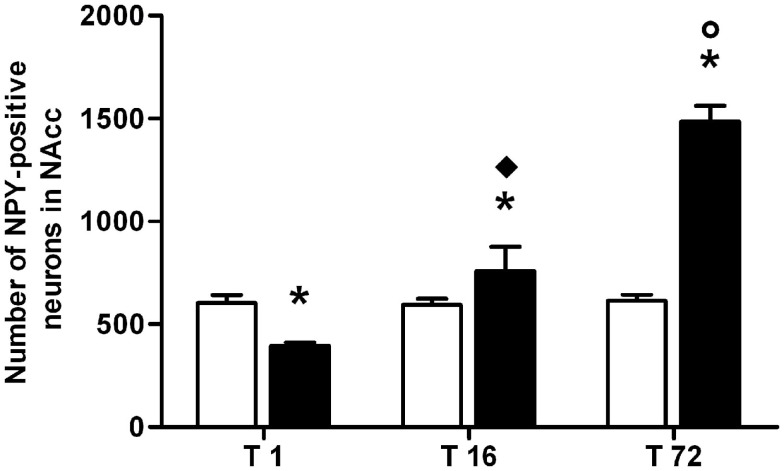
**Average number of neuropeptide Y (NPY)-positive neurons detected in the nucleus accumbens (NAcc) during acetaldehyde intoxication and withdrawal**. Each value represents the mean ± S.D. of 12 sections for each experimental condition. (□) CTR, (■) ACD. *p < 0.001 vs CTR, ^♦^*p* < 0.001 vs T1, ^○^*p* < 0.001 vs T16.

Results from a two-way ANOVA including “ACD treatment” as the between-subjects factor and “time” as within-subjects factor performed on the number of NPY-positive neurons in NAcc, at different time points, showed a significant effect of time, treatment, and their interaction [*F*_(2, 66)_ = 185.97, *p* < 0.0001; *F*_(1,66)_ = 139.60, *p* < 0.0001; *F*_(2, 66)_ = 176.71, *p* < 0.0001]. Bonferroni *post hoc* analysis showed a significant reduction in NPY-positive neurons in ACD group at T1 (*t* = 5.036, *p* < 0.001) and an increase in NPY-positive neurons at T16 (*t* = 3.995, *p* < 0.001) and T72 (*t* = 21.14, *p* < 0.001), when compared to CTR group (Figure 5). NPY expression was also analyzed within ACD group in order to reveal time-related differences in relative NPY-positive neurons during withdrawal. The results of a two-way ANOVA showed a significant effect of time, treatment, and their interaction [*F*_(2, 66)_ = 195.97, *p* < 0.0001; *F*_(1,66)_ = 134.59, *p* < 0.0001; *F*_(2, 66)_ = 176.57, *p* < 0.0001]. Bonferroni *post hoc* analysis showed that NPY expression increased at T16 (*t* = 8.788, *p* < 0.001), with respect to T1, and at T72 (*t* = 17.65, *p* < 0.001) compared to T16 (Figure 6). When NPY expression was evaluated in accumbal sub-regions shell and core, a two-way ANOVA performed, respectively, at T1, at T16, and at T72, including “ACD treatment” as the between-subjects factor and “sub-regional NPY expression” as within-subjects factor showed a significant effect of sub-regional NPY expression [*F*_(1, 44)_ = 199.59, *p* < 0.0001; *F*_(1,44)_ = 16.67, *p* < 0.0002; *F*_(1, 44)_ = 5.11, *p* < 0.028], treatment [*F*_(1, 44)_ = 353.82, *p* < 0.0001; *F*_(1,44)_ = 156.94, *p* < 0.0001; *F*_(1,44)_ = 257.57, *p* < 0.0001] and their interaction [*F*_(1,44)_ = 7.10, *p* < 0.0001; *F*_(1,44)_ = 57.90, *p* < 0.0001; *F*_(1,44)_ = 24.45, *p* < 0.0001]. Bonferroni *post hoc* analysis showed a decrease in the number of NPY-positive cells at T1 in ACD group in shell and core, while an increase in NPY expression was observed at T16 and T72, compared to controls (Table 3). In order to evaluate whether ACD treatment could differentially affect NPY expression in the shell of the NAcc, with respect to core, a two-way ANOVA performed on “sub-regional NPY expression” as the between-subjects factor” and “time” as within-subjects factor, displayed a significant effect of sub-regional NPY expression [*F*_(2,66)_ = 1475.13, *p* < 0.0001], time [*F*_(1,66)_ = 23.86, *p* < 0.0001] and their interaction [*F*_(2,66)_ = 37.10, *p* < 0.0001]. Bonferroni *post hoc* analysis showed that NPY expression decreased at T1 (*t* = 4.036, *p* < 0.001) and increase at T16 (*t* = 4.890, *p* < 0.001) and T72 (*t* = 7.606, *p* < 0.001) in shell when compared to core in ACD-treated rats.

**Table 3:** **Number of NPY-positive neurons expression in nucleus accumbens shell and core, in ACD and in control rats at different times following the last intragastric infusion (T1, T16, T72)**.

Time	Nucleus accumbens sub-regions	CTR	ACD	Statistic	
				*t*	*p*
T1	Shell	265 ± 26.5	158 ± 13.0*	15.18	<0.001
	Core	336 ± 21.0	236 ± 23	11.42	<0.001
T16	Shell	262 ± 30.0	479 ± 47.0*	14.24	<0.001
	Core	300 ± 41.0	278 ± 28.0	3.478	<0.01
T72	Shell	270 ± 96.0	841 ± 103.0*	14.844	<0.001
	Core	343 ± 78.0	645 ± 98.0	7.851	<0.001

Values are mean ± S.D. of 12 sections for each experimental condition. *p < 0.001 vs core.

#### Effects of AM281 on accumbal NPY expression

**Figure 7:**
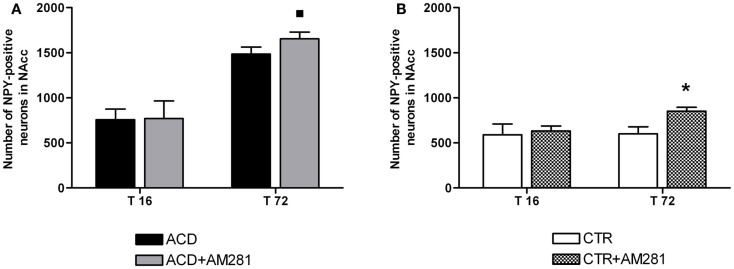
**Effect of AM281 treatment on the number of nucleus accumbens neuropeptide Y (NPY)-positive neurons in ACD rats (A) and in controls (B)**. Each value represents the mean ± S.D. of 12 sections for each experimental condition. *p < 0.001; ^■^*p* < 0.01 vs respective controls.

In order to assess the involvement of CB1 signaling in the modulation of NPY-positive neurons expression in NAcc, statistical analysis by a two-way ANOVA was performed on the effects exerted by the CB1 antagonist AM281 both in ACD group and in controls. Our results showed significant effects of time, treatment, and their interaction [*F*_(1, 44)_ = 489.21, *p* < 0.0001; *F*_(1,44)_ = 6.24, *p* < 0.0163; *F*_(1, 44)_ = 4.46, *p* < 0.043]. Bonferroni *post hoc* analysis displayed that AM281 was able to induce an increase in the number of NPY-positive neurons at T72 (*t* = 3.260, *p* < 0.01) in ACD group compared to respective controls (Figure 7A). Furthermore, when CTR animals received the selective cannabinoid antagonist, statistical analysis performed by a two-way ANOVA including “AM281 treatment” as the between-subjects factor and “time” as within-subjects factor revealed a significant effect of time, treatment, and their interaction [*F*_(1, 44)_ = 25.74, *p* < 0.0001; *F*_(1,44)_ = 41.33, *p* < 0.0001; *F*_(1, 44)_ = 20.69, *p* < 0.0001]. Bonferroni *post hoc* analysis showed that AM281 was able to induce an increase in the number of NPY-positive neurons at T72 (*t* = 7.762, *p* < 0.001) in CTR group, compared to respective controls (Figure 7B).

## Discussion

In the current study, the primary goal was to verify if ACD, the first metabolite of alcohol, is able to produce alterations in NPY protein levels in hippocampus and ventral striatum neurons, as already shown for alcohol (51, 64–66). The major result is that a 4-day long ACD binge treatment modulates NPY expression as a result of ACD intoxication but also as a consequence of early and prolonged, acute withdrawal. In particular, a significant decrease in NPY-positive neurons was observed 1 h after the last ACD infusion both in hippocampus and in NAcc; moreover, 16 and 72 h following the last ACD administration, a relevant increase in NPY expression was reported in the same areas. As recorded following alcohol binge treatment, ACD intoxication produced somatic withdrawal features that were observed at 12 h from the last ACD administration, started to decline at 16 h, and disappeared at 36 h abstinence. ACD-induced physical dependence did not display all the classical signs observed in alcohol withdrawal; indeed, general hyperactivity, irritability, tail tremors, tail stiffness, general tremor, and spasticity were recorded and scored, but they reached a lower severity than in alcohol withdrawal (49, 50, 62); wet dog shakes and spontaneous convulsive seizures were not observed in these experimental conditions. Several factors can contribute to the explanation of this evidence that, as far as we know, has never been reported before. As alcohol, peripherally administered ACD does reach the brain, due to its capability of overwhelming the metabolic barrier constituted by epithelial aldehyde dehydrogenase, a low Km ACD-oxidizing enzyme expressed in the gastrointestinal tract (67, 68). Moreover, high blood ACD concentration can saturate the moderate aldehyde dehydrogenase activity of the BBB capillaries, enter the brain and exert central activity (69–72). Indeed, ACD itself is able to interact with channels and receptors producing relevant alterations in discrete neurotransmitter systems (9, 73–75). Therefore, although ACD does not share the same pharmacodynamic and pharmacokinetic properties of alcohol, its involvement as a mediator of alcohol-induced dependence cannot be ruled out. ACD-induced reduction in NPY expression was observed in all the hippocampal sub-regions, with a prominent effect in DG and CA1 where NPY is expressed in the basket and the granule cells (64). NPY is known to inhibit excitatory transmission by reducing glutamate release (29); indeed, mice lacking NPY are more susceptible to the epileptogenic effect exerted by pentylenetetrazole and kainate (76, 77). In accordance, the reduction in NPY levels observed in this study in the hippocampus, as a consequence of the effect of ACD binge treatment, could be associated to a dampened inhibitory transmission that contributes to the hyperexcitable state that follows physical dependence. The reduction in NPY levels in NAcc following ACD intoxication is consistent with data showing that excessive alcohol drinking behavior is related to lower expression in NPY protein in NAcc in C57BL/6J mice, which innately consume larger amount of alcohol. Conversely, mice showing lower intake of alcohol display higher expression of NPY neurons in the same area. Alcohol drinking behavior has been related to profound modifications in the transductional processes in NAcc neurons that are correlated to lower expression of NPY gene (25). Our data fall into agreement with these findings pointing to a prominent role of NPY in modulating the rewarding, reinforcing, and motivational responses in the mesolimbic system (78, 79). Notably, a dramatic, and time dependent, increase in NPY levels was observed at 16 and 72 h of withdrawal in all the hippocampal sub-regions, primarily in DG and CA1, as well as in NAcc. Alcohol withdrawal is characterized by a great perturbation of the homeostatic systems that in fact leads to the appearance of profound signs of physical dependence. Therefore, it is reasonable to hypothesize that the increase in NPY protein levels in hippocampus, in particular in CA1 and DG, primarily represents a compensatory mechanism, which allows the organism to limit the intensity and duration of hippocampal hyperexcitability. Accordingly, intracerebroventricular administration of NPY has been shown to reduce alcohol withdrawal seizures, after a 4-day alcohol treatment similar to our ACD binge protocol (38). Interestingly, DG is one of the brain regions where neurogenesis of adult neurons occurs, together with the subventricular zone of the lateral ventricles, that in human beings supplies new interneurons to the adjacent striatum (80).

Neurogenesis is a neuronal activity driven process involved in stress-mediated behavioral responses, mood control, and certain forms of learning and memory; NPY has been reported to be a promoter of this form of neuronal plasticity in terms of enhanced proliferation and differentiation in DG neurons in adult mice (40, 41). Hence, the increase in NPY levels, following acute withdrawal observed in this study, could induce hippocampal and subventricular neurogenesis and neural homeostasis as a compensatory mechanism toward ACD-induced loss of inhibitory control and neuronal damage, as also reported following alcohol intoxication (51, 81, 82). Moreover, NPY enhancement in NAcc suggests a direct interaction between dopamine and NPY and in particular the set up of a recovery process in the mesolimbic dopaminergic transmission, so that an increase in NPY levels could boost the activity of TH-positive neurons and increase extracellular dopamine release (83). These findings lead to focus on NPY as a crucial player in the modulation of accumbal dopamine transmission. On the other hand, reports on nigrostriatal regulation in human brain with Parkinson’s disease show that NPY mRNA expression rises as a consequence of a dampened dopaminergic tone (84). The hypodopaminergic state is a neurochemical and functional feature of withdrawal after the chronic exposure to several drugs of abuse, including alcohol (85). Therefore, we hypothesize that the rise in NPY levels in the ventral striatum during early and prolonged acute withdrawal, besides a putative role in promoting neuroplasticity, could contribute to the slow, long transition phase that takes to the reinstatement of the dopaminergic tone, which is compromised during ACD treatment. A very few reports examine the expression of NPY in the striatum following alcohol intoxication, and most of them describe a modulation of NPY in Nacc shell (86). In this study, we observed a modulation of NPY expression in both the accumbal sub-regions, although the increase in NPY levels was prominent in NAcc shell, the accumbal sub-region primarily involved in adaptive neuromechanisms underlying the onset of addiction.

The second major finding of this study is the evidence of an interplay between NPY expression and CB1 signaling in rat hippocampus and NAcc. Endocannabinoids can be released in NAcc (87) and VTA, where they modulate the excitatory and inhibitory inputs that control mesolimbic pathways by acting as retrograde messengers on CB1 receptors (88–92). In this study, the administration of AM281, a selective CB1 antagonist, avoid of aspecific effects, or partial agonist activity produced a significant increase in NPY-positive neurons both in ACD group and in controls. Indeed, injected before the onset of the withdrawal syndrome, AM281 was able to reduce the behavioral signs that follow ACD treatment suspension. This was accompanied by a further increase in the number of NPY-positive neurons both in hippocampus and in NAcc, measured, respectively, at early and prolonged acute withdrawal. Little is known about the functional role of endocannabinoids on NPY system. It has been reported that anandamide and 2-arachidonoylglycerol, through CB1 receptor signaling, dose dependently downregulate NPY mRNA levels (58). The activation of the CB1 signal is associated with the inhibition of PKA and CREB phosphorylation (93). Indeed, the reduction in pCREB could explain the decrease in NPY levels observed, in that NPY gene is a cAMP-inducible element. Moreover, during a binge alcohol treatment, an increase in CREB mRNA and pCREB-IR has been reported at 24 and 72 h withdrawal, when higher NPY expression was also found in the basket cells of the hippocampus (64); this is consistent with our finding and prompts to suggest an inverse correlation between endocannabinoids and NPY expression in the area analyzed. The modulation by AM281 appears to be region- and time-dependent; NPY expression in ACD binge treated rats is increased in NAcc at 3 days of withdrawal, while in the hippocampus it rises at 16 h of withdrawal. At present, this different pattern of expression is difficult to explain; however, a peculiar sensitivity of NPY system to CB1 signaling in NAcc, along with the impact of intracellular and molecular disarrangements linked to withdrawal and the onset of the processes of recovery, cannot be ruled out. In conclusion, the present study shows that complex plastic changes take place in NPY system during ACD binge treatment and subsequent withdrawal in the rat hippocampal formation and NAcc; we hypothesize that ACD binge treatment increases endocannabinoidergic transmission, similar to alcohol (94), thus resulting in a downregulation of NPY system that plays a role in physical dependence and in the onset of withdrawal syndrome. During early and prolonged acute withdrawal, NPY expression progressively rises, likely as a consequence of the decreased endocannabinoidergic tone, thus contributing to the control of neuronal hyperexcitability and of the disarrangement in the mesolimbic system. The pharmacological inhibition of CB1 signaling could be effective in counteracting the neurochemical imbalance associated with ACD and alcohol withdrawal, likely boosting the setting up of homeostatic functional recovery.

## Conflict of Interest Statement

The authors declare that the research was conducted in the absence of any commercial or financial relationships that could be construed as a potential conflict of interest.
